# Prävalenz des „Spitzfußes“ bei stationär behandelten Patienten eines Universitätsklinikums

**DOI:** 10.1007/s00113-025-01582-x

**Published:** 2025-05-06

**Authors:** Alexander Milstrey, Leo-Lion Eisfeld, Jeanette Köppe, Jens Minnerup, Michael J. Raschke, Sabine Ochman, J. Christoph Katthagen

**Affiliations:** 1 Klinik für Unfall‑, Hand- und Wiederherstellungschirurgie, Universitätsklinikum Münster, Münster, Deutschland; 2 Institut für Biometrie und Klinische Forschung, Universität Münster, Münster, Deutschland; 3 Klinik für Neurologie mit Institut für Translationale Neurologie, Universitätsklinikum Münster, Münster, Deutschland

**Keywords:** OSG, Range of Motion, Dorsalextension, Achillessehnenverkürzung, Gangbild, Ankle, Range of Motion, Dorsiflexion, Achilles tendon shortening, Gait pattern

## Abstract

**Hintergrund:**

Die Prävalenz von Spitzfußfehlstellungen ist ein aus Sicht der Autoren unterschätztes medizinisches und sozioökonomisches Problem und bislang unzureichend untersucht. Eine Dorsalextension im oberen Sprunggelenk (OSG) < 10° kann zu Einschränkungen des Gangbildes führen.

**Ziel der Arbeit:**

Die Arbeit sollte die Prävalenz von Spitzfußfehlstellungen anhand der Einschränkung der Dorsalextension im OSG in Grad bei stationär behandelten Patienten erfassen.

**Material und Methoden:**

In dieser Querschnittstudie wurden die aktiven Bewegungsgrade im OSG von 205 Patienten am Universitätsklinikum Münster prospektiv mit einem Goniometer untersucht. Eingeschlossen wurden 136 unfallchirurgische und 69 neurologische Patienten. Der Zusammenhang einer eingeschränkten Dorsalextension zu weiteren klinischen Parametern wurde mittels Chi-Quadrat bzw. Exaktem Test nach Fischer untersucht. Das Signifikanzniveau wurde auf *p* < 0,05 festgelegt.

**Ergebnisse:**

Insgesamt wurden 205 Patienten mit einem Altersdurchschnitt von 59 Jahren eingeschlossen. Bei der Geschlechterverteilung lag eine leichte Prädominanz des männlichen Geschlechtes (55,1 %) vor. Die durchschnittliche Dorsalextension im oberen Sprunggelenk lag rechtsseitig bei 7,03° und linksseitig bei 8,92°. Es wiesen 72,2 % der Patienten eine Dorsalextension im OSG < 10°, 38,1 % eine Dorsalextension < 5° und 16,1 % eine Dorsalextension < 0° auf mindestens einer Seite auf. Es konnte kein relevanter Zusammenhang zwischen Alter, Geschlecht, Dauer des stationären Aufenthalts oder Behandlungsgrund und dem Auftreten einer Einschränkung der Dorsalextension beobachtet werden.

**Diskussion:**

Die Ergebnisse lassen vermuten, dass die Einschränkung der Dorsalextension ein häufiges Problem in unserem Kollektiv von unfallchirurgischen und neurologischen Patienten darstellt. Insbesondere das Vorliegen von > 10 % manifesten Spitzfüßen, die die Neutralposition im OSG nicht erreichen konnten, ist ein relevantes Ergebnis.

**Graphic abstract:**

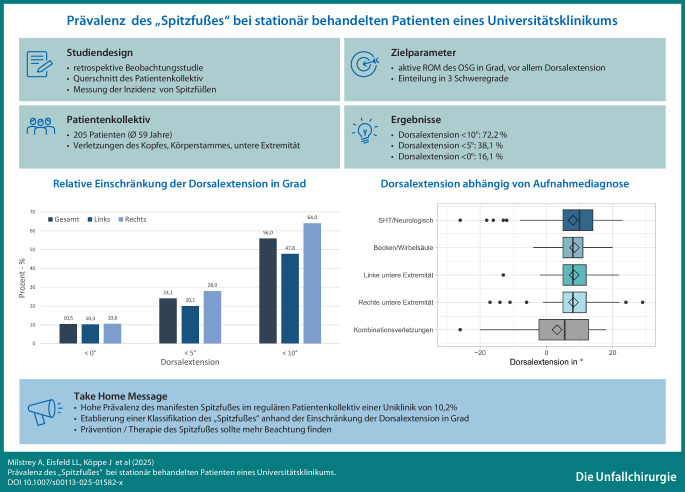

## Abstract (en)

**Background:**

In the authors′ opinion the prevalence of equinus foot deformities is an underestimated medical and socioeconomic problem that has not yet been adequately investigated. A  dorsiflexion in the ankle joint of < 10° can lead to limitations in gait.

**Objective:**

The aim of the present study was to determine the prevalence of equinus foot deformities based on the degree of restriction of dorsiflexion in the ankle joint in hospitalized patients.

**Material and methods:**

In this cross-sectional study the active range of motion of the ankle joint in 205 patients at Münster University Hospital was prospectively examined with a goniometer. Included in the study were 136 trauma surgery and 69 neurology patients. The association of restricted dorsiflexion with other clinical parameters was investigated using the χ^2^-test or Fischer′s exact test. The significance level was set at *p* < 0.05.

**Results:**

In total, 205 patients with mean age of 59 years were included. There was a slight predominance of males (55.1%) in the gender distribution. The average dorsal extension of the ankle joint was 7.03°on the right side and 8.92° on the left side. Of the patients 72.2% had a dorsiflexion in the ankle joint of < 10°, 38.1% a dorsiflexion < 5° and 16.1% a dorsiflexion < 0° on at least one side. A relevant association between age, gender, duration of hospitalization and the reason for treatment with a restriction of dorsiflexion could not be observed.

**Conclusion:**

The results suggest that restriction of dorsiflexion is a frequent problem in our group of trauma surgery and neurology patients. In particular, the presence of a manifest equinus deformity of the foot of > 10% that could not achieve the neutral position is a relevant finding.

## Einleitung

Der Spitzfuß wird von Döderlein definiert als eine funktionelle Beinverlängerung durch eine eingeschränkte Dorsalextension im oberen Sprunggelenk (OSG), die zu einer erhöhten Vorfußbelastung sowie Veränderungen des Gangbildes führt [7, 11]. Hierbei wird die seltenere angeborene Form, am ehesten ausgelöst durch Fehllagen im Uterus (beispielsweise dem Klumpfuß), von der deutlich häufigeren erworbenen Form, beispielsweise nach Trauma, Operationen, oder der spastischen Form bei infantilen Zerebralparesen und Schlaganfällen, abgegrenzt [7, 22]. Nach einem Krankenhausaufenthalt wird die Prävalenz eines nichttraumatischen erworbenen Spitzfußes zwischen 15 und 70 % angegeben [9].

Ein Spitzfuß wiederum kann neben den funktionellen Einschränkungen auch zu sekundären Komplikationen, wie einem Druckulkus im Vorfußbereich, einem Hallux valgus, einer Plantarfasziitis oder einem pathologischen Rückfußvarus/-valgus und somit hohen sozioökonomischen Folgekosten, führen [17, 21].

Die Prävalenz des Spitzfußes ist in der Literatur wiederum nur unzureichend beschrieben, ist jedoch ein essenzieller Parameter in der Beurteilung der Relevanz des Krankheitsbildes [6, 7]. So wird von Döderlein weiterhin auf eine Studie von Bessel-Hagen aus Berlin verwiesen, der im 19. Jh. unter 89.987 Patienten innerhalb von 9,5 Jahren 45 Spitzfüße (0,05 %) diagnostizierte [4, 7]. Einzelne neuere Fallserien mit kleineren Kollektiven aus Patienten mit Fuß- und Sprunggelenkpathologien deuten jedoch auf eine höhere Prävalenz einer eingeschränkten Dorsalextension bis zu 88 % bei 34 Patienten mit Fußschmerzen [6] hin, bzw. dem Vorliegen eines manifesten Spitzfußes mit einer Dorsalextension < 0° bei 10,3 % in einem Kollektiv von Patienten mit Diabetes mellitus [14].

Die Spitzfußfehlstellung wird üblicherweise nicht anhand der Einschränkung der Dorsalextension in Grad eingeteilt, sondern anhand der Ätiologie, Pathogenese oder der Morphologie unterschieden, wie beispielsweise dem Vorliegen eines Rückfuß- oder Vorfußspitzfußes [7]. Eine quantitative Klassifikation abhängig von der Limitation der Dorsalextension in Grad ist nicht bekannt. Eine Dorsalextension im OSG > 10° wird als physiologisch betrachtet [6, 8, 13], wohingegen das Vorliegen eines Spitzfußes meist anhand der Unterschreitung eines Grenzwerts je nach Studie ab einem Wert von 10°- bis 0°-Dorsalextension definiert wird [3, 6, 11–14].

Das Ziel dieser Studie war es daher, die Häufigkeit von Spitzfußfehlstellungen bei stationären Patienten an einem Universitätsklinikum zu untersuchen.

## Material und Methoden

In die vorliegenden Querschnittstudie wurden volljährige, einwilligungsfähige Patienten der Klinik für Unfall‑, Hand- und Wiederherstellungschirurgie sowie der Klinik für Neurologie des Universitätsklinikums Münster eingeschlossen: diese wurden aufgrund von Verletzungen oder Erkrankungen der unteren Extremität, des Rumpfes oder aber des Kopfes bzw. Zentralnervensystems stationär behandelt. Zwischen dem 09.12.2021 und dem 26.01.2023 erfolgte der Einschluss von 136 unfallchirurgischen und 69 neurologischen Patienten. Bei Verletzung eines Sprunggelenks/Fußes oder dem Vorliegen einer Hemiparese erfolgte der Ausschluss der verletzten bzw. paretischen Körperseite (*n* = 37), sodass insgesamt 373 Sprunggelenke in die Studie eingeschlossen werden konnten. Ausschlusskriterium war eine isolierte Verletzung oder Erkrankung der oberen Extremität als Behandlungsgrund. Das Forschungsprojekt wurde durch die Ethikkommission der Ärztekammer Westfalen-Lippe genehmigt (Aktenzeichen 2020-893-f-S).

Eine A‑priori-Power-Analyse wurde mit G*Power (Version 3.1) durchgeführt. Auf der Grundlage von Mittelwerten und Standardabweichungen aus früheren Studien [6] zur Prävalenz des Spitzfußes wurde ermittelt, dass eine Stichprobengröße von 159 Personen die Identifizierung von Unterschieden der Einschränkung der Dorsalextension von 4,4° (bei einer Standardabweichung von 10,9°) mit 99 %iger Power und einem Signifikanzniveau von *p* < 0,05 ermöglichen würde.

**Abb. 1:**
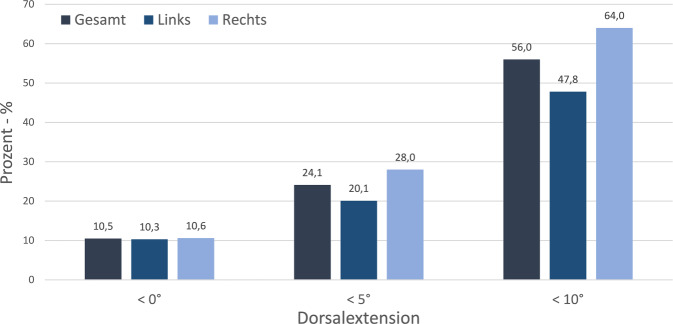
Relative Häufigkeit der Patienten mit Einschränkung der Dorsalextension im oberen Sprunggelenk in Grad, aufgeteilt in 3 Gruppen (< 0°, < 5°, < 10°). Neben der Gesamtzahl wurde die relative Häufigkeit zudem seitenabhängig (links/rechts) untersucht und dargestellt

Die Messung der aktiven Bewegungsgrade entsprechend der Neutral-Null-Methode im oberen Sprunggelenk erfolgte mit einem digitalen Goniometer (Firma Preciva, Shanghai, China) in Rückenlage bei gestrecktem Knie- und Hüftgelenk (Abb. 1). Das Vorliegen eines Spitzfußes wurde anhand der Einschränkung der Dorsalextension im OSG in drei Gruppen unterteilt: 1.) Leichtgradiger Spitzfuß: < 10° Dorsalextension, 2.) Mittelgradiger Spitzfuß: < 5° Dorsalextension und 3.) Schwergradiger/Manifester Spitzfuß: < 0° Dorsalextension.

Korrelationen zwischen Dorsalextension (links/rechts) mit Alter bzw. Liegedauer erfolgten anhand des Spearman-Korrelationskoeffizienten. Unterschiede zwischen den Geschlechtern wurden mittels zweiseitigem Mann-Whitney-U-Test und zwischen dem Behandlungsgrund mittels zweiseitigem Kruskal-Wallis-Test getestet. Alle Analysen sind rein explorativ, *p*-Werte < 0,05 wurden als statistisch auffällig angesehen und entsprechend einer Hypothesengenerierung interpretiert. Es erfolgte keine Adjustierung auf ein multiples Testproblem. Statistische Analysen wurden mit IBM SPSS Statistics for Windows (Version 28.0. IBM, Armonk, NY: IBM Corp) und R Version 4.2.0 (2022-04-22, R Foundation for statistical computing, Wien, Österreich) durchgeführt.

## Ergebnisse

Bei den eingeschlossenen 205 Patienten (113 (55,1 %) männliches und 92 (44,9 %) weibliches Geschlecht) lag der Altersdurchschnitt bei 59,3 Jahren (Standardabweichung ±18,8). Die häufigsten Gründe für die stationäre Behandlung waren in 74 Fällen (36,1 %) ein Schädel-Hirn-Trauma oder eine Erkrankung des Zentralnervensystems, in 94 Fällen (45,9 %) eine Verletzung/Erkrankung der unteren Extremität und in 27 Fällen (13,2 %) eine Verletzung oder Erkrankung des Beckens und der Wirbelsäule. Von den eingeschlossenen 69 neurologischen Patienten wurden 33 aufgrund eines Schlaganfalls sowie 14 aufgrund einer entzündlichen ZNS-Erkrankung behandelt; weitere Aufnahmediagnosen waren M. Parkinson, Epilepsie sowie Schwindel.

Die durchschnittliche maximale Dorsalextension im oberen Sprunggelenk betrug 8,0° (rechts: 7,0°, links: 8,9°, Range −34°–2°). Von den untersuchten Sprunggelenken zeigten 209 (56,0 %) einen leichtgradigen Spitzfuß mit einer maximalen Dorsalextension < 10° (rechts: 121/189, links: 88/184). Bei 90 (24,1 %) der eingeschlossenen Sprunggelenke betrug die maximale Dorsalextension < 5° (rechts: 53/189, links: 37/184). Einen manifesten Spitzfuß mit einer Einschränkung der maximalem Dorsalextension ≤ 0° zeigten 39 (10,5 %) der Sprunggelenke (rechts: 20/189, links: 19/184) (Abb. 1).

**Abb. 2:**
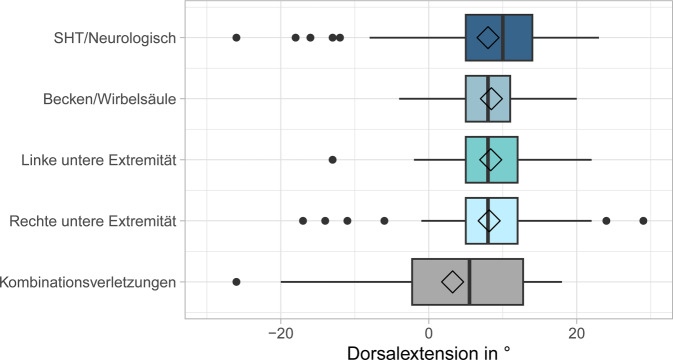
Darstellung der durchschnittlichen Dorsalextension im oberen Sprunggelenk in Grad abhängig vom Aufnahmegrund der Patienten. In den Boxplots liegt der Median jeder Gruppe in der *Mittellinie*, der Mittelwert ist durch die *Raute* dargestellt, die Box ist vom 1. bis zum 3. Quartil gezogen, und die *Linien* präsentieren das Minimum und Maximum jeder Gruppe bis zur 1,5fachen Größe der Box

**Abb. 3:**
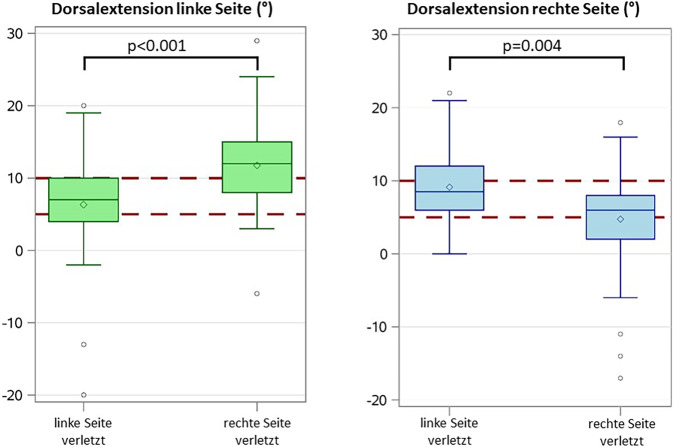
Seitenabhängige Korrelationen zwischen verletzter unterer Extremität und Einschränkung der Dorsalextension in Grad. In den Boxplots liegt der Median jeder Gruppe in der *Mittellinie*, der Mittelwert ist durch die *Raute* dargestellt, die Box ist vom 1. zum 3. Quartil gezogen, und die *Linien* präsentieren das Minimum und Maximum jeder Gruppe bis zur 1,5fachen Größe der Box

Hierbei lag bei 61 (29,8 %) der 205 Patienten ein bilateraler leichtgradiger Spitzfuß, bei 12 (5,9 %) Fällen ein mittelgradiger bilateraler Spitzfuß und in 6 (2,9 %) Fällen ein bilateraler manifester Spitzfuß vor. Es konnten keine relevanten Zusammenhänge zwischen der Dorsalextension und dem Alter, dem Geschlecht sowie die Dauer des stationären Aufenthalts (36 Fälle mit mehrfachen Messungen) beobachtet werden (Tab. 1). Auch zeigten sich seitenunabhängig keine Unterschiede zwischen dem Behandlungsgrund entsprechend der verletzten Körperregion und einer Einschränkung der Dorsalextension (*p* = 0,469; Abb. 2). Unter Berücksichtigung der Seite wurden bei Kombinationsverletzungen reduzierte Dorsalextensionen des rechten Sprunggelenks beobachtet (*p* = 0,009; Tab. 1); links war der Effekt hingegen weniger stark ausgeprägt. Bei Verletzung einer unteren Extremität zeigte sich eine signifikante Einschränkung der Dorsalextension auf der verletzten Seite im Vergleich zur unverletzten Seite (rechts: *p* = 0,004, links: *p* < 0,001; siehe Abb. 3).

**Tab. 1:** Abhängigkeit der Dorsalextension von verschiedenen Patientencharakteristika. Korrelation der Dorsalextension in Grad mit Alter bzw. Dauer des stationären Aufenthalts erfolgte über Berechnung des Spearman Korrelationskoeffizienten mit dazugehörigem *p*-Wert. Unterschiede in der Dorsalextension zwischen Männer und Frauen wurden mithilfe eines zweiseitigen Mann-Whitney-U-Tests und Unterschiede in der Dorsalextension zwischen den Behandlungsgründen mittels Kruskal-Wallis-Test getestet

Variable	Dorsalextension linker Fuß	Dorsalextension rechter Fuß
Alter – Korrelation nach Spearman	R_s_ = −0,159	R_s_ = −0,155
(*p*-Wert)	(*p* = 0,032)	(*p* = 0,035)
Geschlecht – Mittelwert ± Standardabweichung		
Frauen	9,4° ± 6,8°	6,7° ± 8,4°
Männer	8,5° ± 7,9°	7,3° ± 8,2°
(*p*-Wert)	(*p* = 0,266)	(*p* = 0,664)
Behandlungsgrund – Mittelwert ± Standardabweichung		
SHT/Neurologisch	8,8° ± 8,4°	7,2° ± 10,1°
Becken/Wirbelsäule	9,6° ± 4,9°	7,3° ± 4,7°
Linke untere Extremität	7,1° ± 6,6°	9,4° ± 4,8°
Rechte untere Extremität	11,6° ± 6,0°	4,2° ± 7,3°
Kombinationsverletzungen	4,9° ± 11,1°	1,1° ± 15,3°
(*p*-Wert)	(*p* = 0,035)	(*p* = 0,009)

## Diskussion

Die Ergebnisse unserer Studie zeigen eine hohe Prävalenz einer eingeschränkten Dorsalextension < 10° von 56,3 % der untersuchten Sprunggelenke sowie das Vorliegen eines manifesten Spitzfußes in 10,2 % der Fälle. Die Einteilung in drei Gruppen anhand der Einschränkung der Dorsalextension in Grad führte zu einer suffizienten quantitativen Diskrimination. Im Rahmen dieser prospektiven Querschnittstudie konnten von den 205 untersuchten Patienten 373 Sprunggelenke untersucht werden. Diese Studie stellt das bisher größte Patientenkollektiv zur Untersuchung der Spitzfußfehlstellungen dar.

In der Literatur existieren wenige kleinere Fallserien; so konnte DiGiovanni et al. in einem Kollektiv aus 34 Patienten mit Fußschmerzen sowie 34 gesunden Patienten bei extendiertem Kniegelenk eine Einschränkung der Dorsalextension < 10° in 88 % bzw. 44 % finden; eine Restriktion der Dorsalextension < 5° zeigte sich hingegen in 65 % bzw. 24 % der Fälle [6]. Insgesamt sind die Ergebnisse der Studie kongruent zu den Ergebnissen unseres heterogenen Patientenkollektivs mit einer Einschränkung der Dorsalextension < 10° in 72,2 % sowie einer Dorsalextension < 5° in 38,1 % der Fälle. Die Studie von DiGiovanni et al. [6] zeigt einen deutlichen Unterschied in der Inzidenz der Spitzfußfehlstellunggen zwischen den beiden Kollektiven (bestehende Fußschmerzen vs. gesunde Patienten) und verdeutlicht somit den Zusammenhang zwischen eingeschränkter Dorsalextension und deren negativem Einfluss auf das Gangbild bzw. die klinische Relevanz des Krankheitsbildes.

Jastifer und Marston hingegen konnten in einem Kollektiv aus 66 Patienten mit Fußschmerzen sowie 66 gesunden Patienten bei extendiertem Kniegelenk mit einem Goniometer eine durchschnittliche Dorsalextension von 3,6° bei den symptomatischen Patienten bzw. 6,1° bei den gesunden Probanden zeigen [13], hingegen betrug im Kollektiv unserer Studie die durchschnittliche Dorsalextension 8,0°. In der Studie von Jastifer und Marstion erfolgte keine Unterteilung in weitere Gruppen z. B. anhand der Einschränkung der Dorsalextension in Grad. Die Ergebnisse der Messung mit dem Goniometer wurden in der Studie der Messung mit einem speziellen „range of motion device“ mit einer forcierten passiven Dorsalextension durch Applikation eines hohen Dorsalextensionskraftmomentes von 25 Nm gegenübergestellt. Hierdurch konnte die Dorsalextension um 8,0° bzw. 11,1° gesteigert werden [13].

In unserer Studie erfolgte die Messung der aktiven Beweglichkeit des oberen Sprunggelenks ausschließlich mit einem digitalen Goniometer, wohingegen in der Literatur weitere Messmethoden verwendet wurden, beispielsweise mittels visueller Messung, 3D-Ganganalyse oder aber den oben genannten Devices mit forcierter Dorsalextension [5, 12, 13, 17]. Youdas et al. konnten hingegen zeigen, dass die Messung der Beweglichkeit des oberen Sprunggelenks mit einem Goniometer gegenüber der visuellen Schätzung überlegen ist [23].

In unserer Studie erfolgte erstmals die Darstellung der Prävalenz einer Spitzfußstellung in drei Gruppen abhängig von der Dorsalextension in Grad. Vorangegangene Studien definierten das Vorliegen eines Spitzfußes bei Unterschreitung eines bestimmten Grenzwerts der Dorsalextension, meist 0°, 5° oder 10° ohne weitere Unterscheidung zwischen Schweregraden [2, 5, 8, 12, 16]. Einige Studien konnten hierbei eine kompensatorisch vermehrte Vorfußbelastung bei einer Einschränkung < 5° Dorsalextension zeigen [12, 16, 20], wohingegen andere Studien bereits eine leichtgradig vermehrte Vorfußbelastung bei Unterschreitung von 10° Dorsalextension zeigen konnten [3]. Eine Einschränkung der Dorsalextension < 0° wiederum korreliert durch den erhöhten plantaren Druck im Vorfuß signifikant mit dem Auftreten von Erkrankungen wie beispielsweise der Plantarfasziitis oder Fußulzera [18, 19]. Kompensationsmechanismen einer eingeschränkten Plantarflexion sind hauptsächlich im Bereich des Kniegelenks lokalisiert mit Kniegelenkflexion und -extension während des „mid-stance phase“ [1, 10, 15]. In der Standphase zeigten sich biomechanisch eine vermehrte Hüft- und Kniegelenkextension [10]. Die Grenze zur signifikanten Veränderung der Kinematik und Kinetik des Gangbilds liegt bei 10° Plantarflexion.

Die Darstellung der Prävalenz mit Unterscheidung in drei verschiedene Gruppen bietet erstmalig die Möglichkeit, quantitative Rückschlüsse auf den Schweregrad einer eingeschränkten Dorsalextension zu ziehen. Hierbei sollte lediglich die schwergradige Form mit einem Nichterreichen der Neutralposition im oberen Sprunggelenk als manifester Spitzfuß definiert werden.

Die vorliegende Studie beinhaltet mehrere Limitationen. Durch das Design der Studie lassen sich keine Rückschlüsse auf die Ätiologie der Spitzfußfehlstellung bzw. den Zeitpunkt der Entstehung ziehen. Zudem wurde die Dorsalextension im OSG ausschließlich in Rückenlage bei gestrecktem Kniegelenk gemessen, sodass durch den Zug des Musculus gastrocnemius möglicherweise die Dorsalextension bereits gehemmt wurde. Hierdurch konnten jedoch wiederum Patienten mit Verletzungen im Bereich des Kniegelenks in die Studie eingeschlossen werden. Zudem erfolgte die Messung kongruent zu den vorangegangenen Studien, sodass die Vergleichbarkeit unserer Studie verbessert wurde. Das vorliegende Patientenkollektiv aus unfallchirurgischen und neurologischen Patienten kann einen Selektionsbias darstellen, da andere Patientengruppen möglicherweise ein geringeres Risiko für eine Einschränkung der Dorsalextension haben, beispielsweise bei einer isolierten Verletzung im Bereich der oberen Extremitäten. Aufgrund des Studiendesigns sind zudem keine Rückschlüsse auf das funktionelle Outcome bzw. die Persistenz der Spitzfußfehlstellung möglich; hier sollten künftige Studien die langfristige Entwicklung untersuchen.

## Fazit für die Praxis

Die hohe Prävalenz von Spitzfußfehlstellungen in unserer Studie legt nahe, dass dieses Problem in der klinischen Praxis oft unterschätzt wird. Die Einschränkung der Dorsalextension kann erhebliche Auswirkungen auf das Gangbild und die Lebensqualität der Patienten haben und ist mit sekundären Komplikationen wie Druckulzera, Hallux valgus und Plantarfasziitis verbunden. Daher sollten präventive und therapeutische Maßnahmen, wie physiotherapeutische Interventionen und ggf. Hilfsmittelversorgung, in Betracht gezogen werden.

## Data Availability

Die Daten, auf denen die Ergebnisse dieser Studie beruhen, sind aus Gründen des Datenschutzes nicht öffentlich zugänglich und können auf begründete Anfrage beim entsprechenden Autor verschlüsselt angefordert werden.
